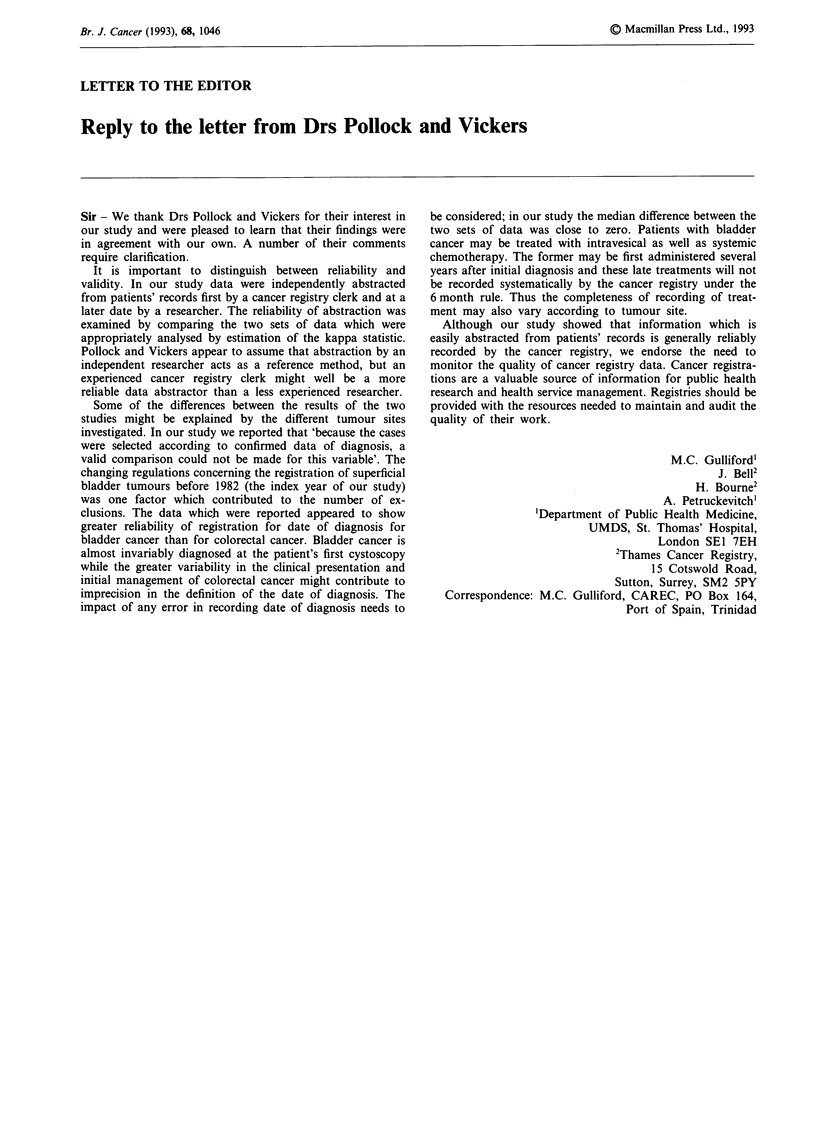# Reply to the letter from Drs Pollock and Vickers

**Published:** 1993-11

**Authors:** M.C. Gulliford, J. Bell, H. Bourne, A. Petruckevitch


 © Macmillan Press Ltd., 1993

LETTER TO THE EDITOR

### Reply to the letter from Drs Pollock and Vickers

**Sir** – We thank Drs Pollock and Vickers for their interest in our study and were pleased to learn that their findings were in agreement with our own. A number of their comments require clarification.

It is important to distinguish between reliability and validity. In our study data were independently abstracted from patients' records first by a cancer registry clerk and at a later date by a researcher. The reliability of abstraction was examined by comparing the two sets of data which were appropriately analysed by estimation of the kappa statistic. Pollock and Vickers appear to assume that abstraction by an independent researcher acts as a reference method, but an experienced cancer registry clerk might well be a more reliable data abstractor than a less experienced researcher.

Some of the differences between the results of the two studies might be explained by the different tumour sites investigated. In our study we reported that 'because the cases were selected according to confirmed data of diagnosis, a valid comparison could not be made for this variable'. The changing regulations concerning the registration of superficial bladder tumours before 1982 (the index year of our study) was one factor which contributed to the number of exclusions. The data which were reported appeared to show greater reliability of registration for date of diagnosis for bladder cancer than for colorectal cancer. Bladder cancer is almost invariably diagnosed at the patient's first cystoscopy while the greater variability in the clinical presentation and initial management of colorectal cancer might contribute to imprecision in the definition of the date of diagnosis. The impact of any error in recording date of diagnosis needs to be considered; in our study the median difference between the two sets of data was close to zero. Patients with bladder cancer may be treated with intravesical as well as systemic chemotherapy. The former may be first administered several years after initial diagnosis and these late treatments will not be recorded systematically by the cancer registry under the 6 month rule. Thus the completeness of recording of treatment may also vary according to tumour site.

Although our study showed that information which is easily abstracted from patients' records is generally reliably recorded by the cancer registry, we endorse the need to monitor the quality of cancer registry data. Cancer registrations are a valuable source of information for public health research and health service management. Registries should be provided with the resources needed to maintain and audit the quality of their work.

M.C. Gulliford[1]
J. Bell[2]
H. Bourne[2]
A. Petruckevitch[1]
[1]Department of Public Health Medicine,
UMDS, St. Thomas' Hospital,
London SE1 7EH
[2]Thames Cancer Registry,
15 Cotswold Road,
Sutton, Surrey, SM2 5PY
Correspondence: M.C. Gulliford, CAREC, PO Box 164,
Port of Spain, Trinidad